# Association of perceived stress and self-control with health-promoting behaviors in adolescents: A cross-sectional study

**DOI:** 10.1097/MD.0000000000011880

**Published:** 2018-08-24

**Authors:** Na-Gyeung Kang, Mi-Ae You

**Affiliations:** a College of Nursing; b College of Nursing and Research Institute of Nursing Science, Ajou University, Suwon, South Korea.

**Keywords:** adolescents, health behavior, health promotion, self-control, stress

## Abstract

Health-related habits acquired during adolescence are strongly related to health problems and behaviors in adulthood. Understanding the health-promoting behaviors of adolescents might help in efforts to encourage them to form lifelong healthy habits, which in turn would have wide-ranging benefits for their quality of life. This study was conducted to examine the associations of perceived stress and self-control with health-promoting behaviors in adolescents. The participants were 292 adolescents (136 boys, 156 girls) ages 15 to 18 years who were asked to complete a self-administered questionnaire. Hierarchical multiple linear regression analysis revealed that middle school students, higher economic status, subjective health status, and self-control were significant influencing factors of health promoting-behaviors. The total explanatory power of these associations was 23.9%. This finding suggests that schools and communities should take an interest in adolescents’ health-promoting behaviors and develop proactive programs that promote the practice of these behaviors.

## 1 Introduction

Adolescence is the transitional period between childhood and adulthood wherein individuals experience rapid physical and psychosocial change.^[1]^ It represents a key maturational stage in which individuals tend to develop their attitudes and behaviors related to healthy lifestyles.^[2–4]^ This makes it an important stage for the prevention of chronic illnesses in adulthood, which often stem from lifestyle habits formed in early childhood and adolescence.^[5,6]^

Health-promoting behavior, defined as acting to take control over and responsibility for the maintenance of one's health status, is necessary for cultivating the ability to maintain one's own health.^[2]^ High school is a risky period for the development of poor lifestyles or behaviors that can lead to chronic illnesses and influence health in adulthood.^[7,8]^ In South Korea, the fierce competition for university admission among high school students has caused students, their families, and schools to largely neglect the issue of health-promoting behaviors; particularly, there are presently rather few health promotion programs in schools or targeting adolescents.

According to the results of a health examination survey among middle and high school students across South Korea,^[9]^ the obesity rate in adolescents appears to increase with age, with the highest rates being observed in 17-year-old males and females (22.6% and 15.3%, respectively). Furthermore, a 2017 online survey^[10]^ on adolescent health behaviors revealed that the practice rate of aerobic physical activity in adolescents was low, with only about 1 in 5 male students (19.5%), and 1 in 13 female students (7.5%) engaging in such activities. Among male high school students, the average daily time spent sedentary while studying during weekdays and weekends was 8.4 hours (506.4 minutes) and 4.6 hours (273.9 minutes), respectively. In addition, around 30.5% of high school students regularly miss breakfast, and this rate appears to be increasing annually; the consumption rate of fast food and carbonated drinks is also increasing.

These alarming statistics have inspired schools in South Korea to begin carrying out health promotion campaigns, such as school meal improvements, dental health programs, health education, and school environment improvements. Nevertheless, even as adolescents’ physical constitution grows, their physical strength declines.^[11]^ The prevalence of excessive internet and smartphones use in adolescents is also increasing, which has been associated with worsening behavior and increasing psychosocial problems, including affective disorders such as depression and anxiety, suicide, school violence, addiction problems such as smoking and drinking, running away from home, dropping out of school, and engaging in delinquent behavior at school.^[2,12]^ Therefore, health promotion programs and campaigns need to make more effort to improve health in adolescents.

The physical, mental, and psychological changes during adolescence are characterized by the developmental task of forming an identity, cause individuals to arguably experience more stress during adolescence than during any other developmental period.^[12,13]^ In South Korea, 32.3% of male high school students and 47.6% of female high school students perceived themselves to be under an enormous or significant amount of stress.^[10]^ Other sources of stress in adolescents are excessive studying and an education system that focuses on passing the increasingly competitive college entrance examinations.^[14]^ These stressful situations can have a negative effect on mental health and lifestyle habits in adolescents.^[15,16]^ For example, higher perceived stress in adolescents is associated with less frequent engagement in health-promoting behaviors, greater consumption of foods high in sugar, and more irregular meals.^[3,14,17]^ High perceived stress might cloud judgement in a similar way as would being in a dangerous situation, leading to less frequent engagement in health behaviors, or even stopping engagement in these behaviors entirely.^[3]^

Self-control is another factor that predicts healthy lifestyle habits.^[18]^ It is defined as the ability to resist temporary impulses in order to achieve larger and more long-term goals, or the ability to resist behaviors that provide instant gratification.^[19]^ Inadequate self-control is linked to behavioral and impulse-control problems.^[20]^ Previous studies have reported that adolescents with higher self-control more frequently engage in physical activity^[18,21]^ and practice healthy eating habits, have a lower body mass index (BMI),^[21]^ and engage in less sedentary behavior.^[22]^ Unfortunately, in South Korea, researchers have primarily examined self-control in the context of internet gaming addiction in adolescents,^[23]^ problem behaviors, or impulse shopping^[12]^; no studies appear to have been conducted on the correlation of health promotion behaviors in adolescents.

Adolescence is a critical period for the formation of attitudes towar health, which in turn strongly influence engagement in healthy and unhealthy behavior.^[3]^ Because such health-related behaviors are associated with health status and quality of life in adulthood, it is essential that adolescents make habits of those that promote health. The purpose of the present study is to provide baseline data for the development of health-promoting behavior programs that seek to habituate such behaviors in adolescents, by determining the associations of perceived stress and self-control with health-promoting behaviors in adolescents.

## 2 Methods

### 2.1 Study design and subjects

A cross-sectional, descriptive study design was used. The subjects were a random sample of students currently enrolled in a middle or high school located in 1 of 3 cities in a province. The minimum number of research subjects was calculated using G∗power 3.1 Program.^[24]^ On the basis of a significance level of 0.05 for a multiple regression analysis, a statistical power of 80%, medium effect size of 0.15, and 12 independent variables, a minimum of 127 subjects was needed. After accounting for 20% the dropout rate, 140 boys and 160 girls, respectively, were surveyed; however, only 292 were included in the final analysis after excluding inadequate responses. The average age of subjects was 16.6 years, and 149 subjects were girls (51.0%). Two-hundred fifty subjects (85.6%) had a normal BMI, 22 (7.5%) were underweight, and 20 (6.8%) were above overweight. As for subjective health status, 163 (55.8%) said “good.” One hundred thirty-one (44.9%) acquired their health information through television (Table 1).

**Table 1:**
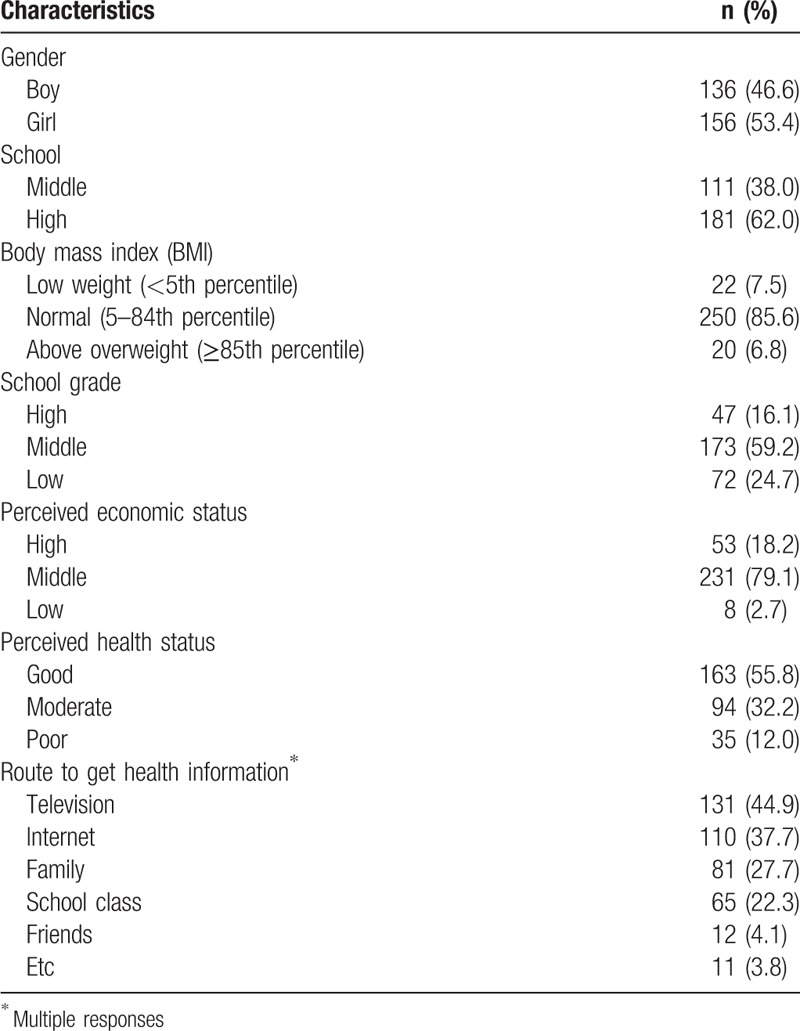
General characteristics of subjects (N = 292).

### 2.2 Measures

#### 2.2.1 Perceived stress

To evaluate perceived stress, we used the stress questionnaire from the Korean Youth Panel Survey (KYPS),^[25]^ which consists of 16 items on individuals’ relationship with their parents, academic performance, relationships with friends, appearance, and substance use. Each item is rated on a 5-point scale ranging from 1 (“never”) to 5 (“always”). Higher scores indicate higher perceived stress. The Cronbach α was 0.90 at the time of development, and 0.85 in the present study.

#### 2.2.2 Self-control

We used the self-control scale developed by Nam and Ok,^[26]^ which consists of 20 items, including 10 assessing the degree to which one seeks long-term satisfaction and 10 assessing the degree to which one seeks immediate satisfaction. Each item is rated on a 5-point scale ranging from 1 (“not at all”) to 5 (“very much so”). Higher scores indicate higher self-control. The Cronbach α was 0.78 in a previous study^[26]^ and 0.76 in the present study.

#### 2.2.3 Health-promoting behavior

We used the short-form Adolescent Health Promotion Scale (AHP-SF) developed by Chen et al^[27]^ to evaluate health-promoting behavior. This tool comprises 21 items in 6 subscales, including nutrition, social support, health responsibility, life appreciation, exercise, and stress management. Each item is rated on a 5-point scale ranging from 1 (“not at all”) to 5 (“always”). Higher scores indicate a higher frequency of practicing health-promoting behaviors. The Cronbach α was 0.91 at the time of the tool's development, and 0.86 in the present study.

### 2.3 Data collection procedure

This study received approval from an institutional review board (AJIRB-SBR-SUR-16-175) before data collection began. The study was conducted from June to July 2016 in middle schools and high schools located in a province.

Cooperation from the principals, teachers in charge, and school nurses of each of the randomly selected schools was obtained after explaining the content and purpose of this study. The school nurses of the selected schools recommended classes that could participate, which ultimately included 2 classes each from grades 2 and 3 of middle school, and 4, 4, and 2 classes from grades 1, 2, and 3 of high school, respectively. The researcher then visited each of the recommended classes to obtain subjects’ consent for participation as well as explain the study's purpose, procedures, and content. Research newsletters and consent forms to home for participation agreement were distributed to the students of all selected classes after the researcher explained that the data would be used only for research purposes that participants could withdraw at any time without any penalty and that participation should be voluntary. We recruited students who submitted written consent with signature of parents and student. Participation consents were collected over 3 days and delivered to the researchers by school nurses.

Subsequently, the surveys were distributed in sealed envelopes in consideration of class schedules with the help of the teachers in charge of the classes that agreed to participate. The surveys were completed by students themselves and submitted anonymously in sealed envelopes to maintain the anonymity and privacy of the subjects. A small present was provided as a token of gratitude to students who participated in the survey.

### 2.4 Data analysis

The general characteristics of the adolescents, levels of perceived stress, self-control, and health promotion behaviors were calculated for errors, percentages, averages, and standard deviations. BMI was calculated as the weight (kg) divided by the square of the height (m^2^) and evaluated from the BMI percentile chart values for children and adolescents.^[28]^ Participants were classified as being of normal weight (< 85th percentile), at risk for overweight (85th to < 95th percentile), or overweight (≥95th percentile), and classified into normal weight (< 85th percentile) and overweight (≥85th percentile) adolescents.

Differences in the health promotion behaviors according to subjects’ general characteristics were analyzed using the independent *t* test and 1-way analysis of variance, and Scheffé test was used for the post hoc analysis. The relationships between perceived stress, self-control, and health promotion behaviors were analyzed via Pearson correlation coefficient. Subsequently, a hierarchical multiple regression was conducted to better analyze the independent factors related to health-promoting behaviors.

Before the multiple regression analysis, we determined whether the basic assumptions of this analysis had been satisfied. The correlations between the independent variables were 0.15 to 0.33; as none were over 0.60, the independent variables were deemed to be independent. Furthermore, the tolerance limit was consistently over 0.1 (at 0.15–0.82) and the variance inflation factors were lower than the standard of 10 (at 1.21–6.65), thus verifying that there were no issues of multicollinearity. The assumptions of the residuals were also all satisfied (including normality, homoscedasticity, and linearity). As for verification of autocorrelations, the results of the Durbin–Watson test were close to 2 (at 2.03), which indicated no autocorrelation, and the maximum value of Cook distance did not exceed 1.0 (at 0.04), verifying that there were no outliers. Significance was set at 0.05 for all statistical tests. All *P* values were 2-sided, and *P* <.05 was considered statistically significant. Statistical tests were performed using IBM SPSS Statistics 23.0 (Datasolution Inc.).

## 3 Results

### 3.1 Descriptive statistics of perceived stress, self-control, and health-promoting behaviors

Subjects’ mean perceived stress score was 42.60 (SD = 10.41) out of a possible 85. The mean self-control score was 70.91 (SD = 7.55), out of a possible 100. Finally, the mean score for health-promoting behaviors was 64.40 (SD = 11.80) out of a possible 105 (Table 2).

**Table 2:**
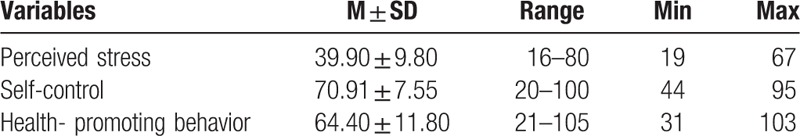
Levels of perceived stress, self-control, and health-promoting behavior.

### 3.2 Differences in health-promoting behaviors according to general characteristics

Statistically significant differences in health-promoting behaviors were observed according to school classification (*t* = 2.63, *P* = .009), grades (*F* = 6.99, *P* = .001), subjective economic status (*F* = 7.31, *P* = .001), and subjective health status (*F* = 8.04, *P* < .001). High school students engaged in more health-promoting behaviors than did middle school students. Students who were middle-ranked in terms of school grade engaged in more health-promoting behaviors than lower-ranked students. Furthermore, higher-ranked students engaged in more health-promoting behaviors than did middle-ranked students. Students with a middle economic status performed more health-promoting behaviors than did those with a low economic status, and the same was true of students with a high economic status than those with a middle status. Students with a normal subjective health status engaged in more health-promoting behaviors than did those who indicated that they had a poor status, and the same was true of students who reported a good subjective health status compared with those who reported a normal status (Table 3).

**Table 3:**
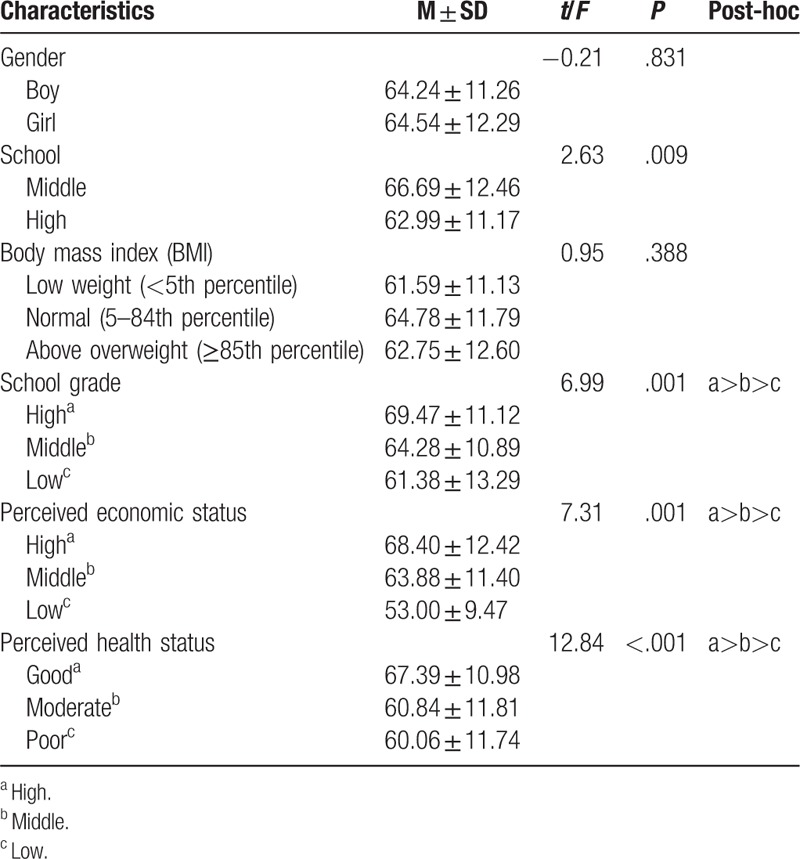
Differences in health-promoting behavior according to general characteristics (N = 292).

### 3.3 Relationship between perceived stress, self-control, and health-promoting behaviors

Statistically significant negative correlations were found between health-promoting behaviors and perceived stress (*r* = −0.15, *P* = .012), as well as between perceived stress and self-control (*r* = −0.33, *P* < .001). Health-promoting behaviors also showed a significant positive correlation with self-control (*r* = 0.33, *P* < .001).

### 3.4 Independent factors related to health-promoting behaviors

Results for a 2-stage hierarchical multiple linear regression analysis of the factors related to health-promoting behaviors are provided in Table 4. First, in step 1, the general characteristics that exhibited statistically significant correlations with health-promoting behaviors were entered. In step 2, the net effects of stress and self-control were evaluated while controlling general characteristics.

**Table 4:**
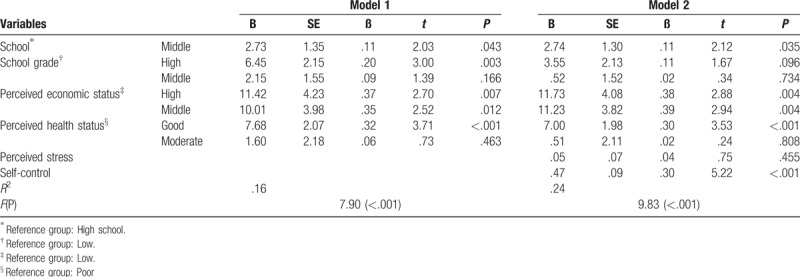
Hierarchical multiple regression analysis of health-promoting behavior.

In step 1, after processing the dummy variables, school classification (middle/high), grades, economic status, and subjective health status were inserted. Middle school (β = 0.11, *P* = .043), high-rank grades (β = 0.20, *P* = .003), high economic status (β = 0.37, *P* = .007) and normal economic status (β = 0.35, *P* = .012), and good subjective health status (β = 0.11, *P* < .001) were revealed to be statistically significant influencing factors, explaining 16.3% of the variance in health-promoting behaviors. In step 2, inserting stress and self-control led to an increase of 7.6% in the variance explained compared with step 1 (for a total of 23.9%). In this analysis, middle school (β = 0.11, *P* = .035), high economic status (β = 0.38, *P* = .004) and middle economic status (β = 0.39, *P* = .004), good subjective health status (β = 0.30, *P* < .001), and self-control (β = 0.30, *P* < .001) were significant influencing factors.

## 4 Discussion

The results revealed that being in middle school students and having higher economic status, better subjective health status, and higher self-control were influencing factors of health-promoting behaviors. The significant relationship between self-control and health-promoting behaviors aligns with the results of a previous study,^[29]^ indicating that higher self-control was associated with increased consumption of fruits and vegetables, and reducing consumption of fast food. Adolescents with high self-control were also reported to have a healthier lifestyle, spend less money on unhealthy foods and drinks, have a lower BMI,^[30]^ and engage in more healthy behaviors such as physical activity.^[18]^ However, despite the importance of healthy nutrition and exercise during adolescence, we were unable to find any previous studies on self-control and health-promoting behaviors in Korean adolescents. Accordingly, replication studies are needed to confirm this relationship.

People with high self-control are able to inhibit or change their inner responses and refrain from engaging in undesirable behaviors.^[30]^ Self-control is strongly influenced by parental childrearing style.^[12,26]^ More specifically, active communication between parents and children is believed to promote children's self-control and help parents recognize problems that the child might be experiencing; this is attributed to the high level of supervision that occurs during such communication.^[26]^ Accordingly, parent education on child-rearing methods might be helpful for improving self-control, which in turn could encourage adolescents’ engagement in health-promoting behaviors. It would also be helpful to directly target self-control in adolescents via reinforcement programs, as this could promote their use of desirable health behaviors.

Among the general characteristics of the subjects, school classification (middle and high), economic status, and subjective health status were significant factors associated with health-promoting behaviors. Specifically, middle school students performed more health-promoting behaviors than did high school students. This might be because high school students consider studying to be a higher priority than health, as they must prepare for college entrance examinations. As for economic status, the results in this study coincide with those of previous studies,^[31–33]^ wherein adolescents with a low socioeconomic status reported poorer nutritional habits and less exercise than did adolescents with a higher socioeconomic status.^[32]^ Accordingly, schools and communities should preferentially take interest in low-income adolescents, and might strive to promote their participation in sports teams as an after-school activity in order to help practice health-promoting behaviors. The results concerning subjective health status are consistent with the results of a previous study on high school students.^[5]^ Another study showed that adolescents who participate in physical activity tend to have a more positive view of their own health status than do adolescents who do not participate in physical activity.^[34]^ Health concern of student was reported as an influencing factor on health-promoting behaviors.^[8,35]^ Therefore, it might be possible to improve students’ health status by activating of health education on school, which might increase their interest of health.

A significant inverse correlation between perceived stress and health-promoting behaviors in adolescents was observed; however, this association was not significant in the regression analysis. This contradicts a previous study showing a significant association between these variables,^[3]^ as well as a study showing that adolescents who frequently miss breakfast because of late bedtimes from studying and early school start times tend to have a higher dependence on snacks and eating out, and that adolescents with higher levels of stress tend to have greater consumption of sugary foods.^[14]^ Other studies have shown that higher perceived stress was associated with more irregular meals, increased consumption of fast foods (which are high in calories and low in nutritional value), and decreased consumption of health foods such as fruits and vegetables.^[17,36]^ Accordingly, continued research on the effects of stress on the health promoting behaviors of adolescents is needed.

A limitation of this study is that it used a convenience sample of middle schools and high schools in 1 region; therefore, the results cannot be generalized to such schools throughout South Korea. Furthermore, the results are based on self-reports by adolescents, so future studies should use more detailed and objective data on physical activity and eating habits, as well as test the possible relations of peer group, family, and community factors with health-promoting behavior in adolescents. Nevertheless, to our knowledge, the present study is the first study to investigate the association of perceived stress and self-control with health-promoting behaviors in Korean adolescents. This study is meaningful in identifying the importance of self-control to enhance health-promoting behaviors of adolescents. Health care provider needs to assess the adolescent's level of self-control when planning interventions and to give more attention in health of low-income adolescents. Knowledge of this relationship can help health care providers develop programs to increase the health-promoting behaviors in adolescents, as well as provide baseline data for future studies.

## 5 Conclusion

Adolescence is a time wherein individuals can lay the foundation for healthy living through proactive health management. Health-related habits acquired during adolescence are strongly related to health problems and behaviors in adulthood. Therefore, understanding the health-promoting behaviors of adolescents might help in efforts to encourage them to form lifelong healthy habits, which in turn would have wide-ranging benefits for their quality of life. Health care providers are in a position to encourage the health promotion of adolescents through mediation, such as by assessing the lifestyle habits of adolescents while promoting positive behavior and reducing negative behavior. The results of the study revealed that being in middle school and having higher economic status, subjective health status, and self-control are associated with more frequent practice of health-promoting behaviors. On the basis of these results, schools and communities can devise better programs aimed at encouraging adolescents to practice such behavior; in particular, they can focus on boosting self-control. In addition, policies focusing on low-income adolescents are particularly needed to help them recognize health as a priority.

## Author contributions

**Conceptualization:** Mi-Ae You, Na-Gyeung Kang.

**Data curation:** Na-Gyeung Kang.

**Funding acquisition:** Mi-Ae You.

**Methodology:** Mi-Ae You, Na-Gyeung Kang.

**Writing – original draft:** Na-Gyeung Kang, Mi-Ae You.

**Writing – review & editing:** Mi-Ae You.
